# Digitalisation of municipal healthcare collaboration with volunteers: a case study applying normalization process theory

**DOI:** 10.1186/s12913-021-06429-w

**Published:** 2021-05-01

**Authors:** Erica Fredriksen, Elin Thygesen, Carl E. Moe, Santiago Martinez

**Affiliations:** 1 Faculty of Health and Sport Sciences, University of Agder, PB 422, 4604 Kristiansand, Norway; 2 Faculty of Social Sciences, University of Agder, PB 422, 4604 Kristiansand, Norway

**Keywords:** Volunteers, Coordination, Collaboration, Healthcare, Digital system, Implementation

## Abstract

**Background:**

Increasing use of volunteers in healthcare requires structured collaboration between healthcare services and volunteers. The aim of this research was to explore critical issues and strategies in the implementation process of a digital solution for collaboration with and coordination of volunteers in municipal healthcare services.

**Methods:**

Qualitative data collection was used to study implementation of a digital system for collaboration with volunteers in three Norwegian municipalities. Three rounds of interviews were conducted with healthcare employees from a volunteer centre and from municipality healthcare units in three municipalities: before implementation, and 6 and 12 months after deployment. Observations of healthcare employees training and use of the system were also done.

**Results:**

An inductive analysis resulted in eleven themes that were grouped based on the four constructs of the normalisation process theory (NPT), plus two themes that fall outside those constructs. Coherence (understanding of the intervention) was high among the employees prior to the intervention. They expected the system to meet several of their needs and increase efficiency, structure and overview. In addition, they expected the system to benefit recruitment strategies along with their matching processes. Cognitive participation (engagement and commitment towards the intervention): employees from two of the municipalities reported absence of leadership and management guidance during the process, management of expectations and clarification of their roles. In the third, there was high engagement and management involvement in the implementation process. Collective action (whether the intervention is carried out): the employees reported time-consuming preparations. Engagement varied between the municipalities. There was a lack of commitment in two due to ongoing reorganisation, in these, the system was partly or not implemented. The third municipality implemented and fully piloted the system. Reflexive monitoring (appraisal towards the system and its impact on practice): the employees learned throughout testing of the system and realised that there were several benefits that could improve their working routines.

**Conclusion:**

Crucial aspects for implementation of the digital tool for collaboration with volunteers include having structure “in place”, establishing policies for involving volunteers, defining clear roles and expectations and involving management and key people (“champions”) to drive the implementation.

**Supplementary Information:**

The online version contains supplementary material available at 10.1186/s12913-021-06429-w.

## Background

The work of volunteers is a necessary contribution to healthcare services because of current trends in the ageing population and pressure on welfare systems as tighter budgets [1]. With the recent increased use of volunteers in the healthcare services in Norway [2], there is a need to study their coordination and management with existing local institutions [3, 4]. However, studies on how healthcare employees coordinate volunteers have found that this coordination is based on informal contact, unsystematic processes and unsecure storage of documentation [3, 5–7]. Hence, there is a need to find new flexible solutions and improve practices that meet these challenges [1, 8].

Other studies have shown that technology solutions may contribute to the coordination of volunteers by supporting tasks in the process, such as recruitment, matching, coordination, overview and statistics [9, 10]. Technology solutions for administrative tasks and coordination exist, but they are often designed for large, professional companies and organisations [11]. However, much of the coordination of volunteers takes place at lower levels in organisations or in the public sector, where there is less need for large, complex systems because few volunteers are handled by personnel with low or moderate levels of technology expertise [10]. Many of the existing technology solutions used by the volunteer sector have been self-created and either do not adequately meet their coordination needs [10] or provide health service solutions to meet them. The reasons described above imply that new tools are needed to fulfil these unmet needs. Thus, in recent years, technological solutions specifically designed for the coordination of volunteers have entered the market [9, 12].

Several challenges associated with the implementation of new technology solutions [13] can be found in the healthcare sector [14]. Additional challenges arise when a system for cooperation between different healthcare organisations or between healthcare and volunteer organisations is implemented. A successful implementation process is vital for healthcare organisations [15]. Therefore, there is a need for additional research in this area [16], especially related to interorganisational cooperation with organisations outside of healthcare services, such as volunteer organisations.

The interconnections between information technology, organisational structures and processes are complex [17], and this may explain the gap in research on the implementation of technology to support collaboration between municipal healthcare and volunteer organisations. We address this gap by studying the implementation of a digital tool for the coordination of volunteers in healthcare services.

The tool was an off-the-shelf solution, but offered some possibilities for adaptation to the different entities. In this tool, volunteers can be assigned to one-to-one tasks or events that involve patients. Volunteers can easily sign up, sign in, subscribe to tasks or unregister. The tool gives an overview of events, tasks and available volunteers. Furthermore, it provides secure storage for documentation [3]. In this process, the challenges related to coordination of volunteers and implementation and application of information systems are common, and as there is a research gap, our study will be of interest to the research community. Practice, especially organisations that coordinate volunteers, can benefit from this research and find new ways to collaborate between volunteer organisations, as it provides insight into the implementation of technology in municipal settings over time.

The system was implemented in three municipalities in Southern Norway and was designed to support collaboration between healthcare services and the volunteer centre regarding the coordination of volunteers. The research question addressed in the current study was:

*What are the critical issues and potential strategies in the implementation process of a digital solution for the collaboration with and coordination of volunteers in municipal healthcare services?*

The findings of the analysis of the implementation were grouped based on the normalisation process theory (NPT) [18]. The NPT is a framework that can help in understanding the process of complex interventions in healthcare services and interventions consisting of behavioural, technological and organisational components [18]. The NPT framework focuses on phenomena that are the products of cooperation and collective activities but that are experienced and explained by the individual participants [18]. According to the NPT, the factors that promote or inhibit implementation can be explained by the following four constructs: coherence, cognitive participation, collective action and reflexive monitoring [18].

Other studies have used the NPT to study the implementation of technology in healthcare [14]. These studies have explored the implementation of an electronic health record in a maternity unit [13], studied the implementation of monitoring technologies in care homes for people with dementia, and [19] carried out a systematic review of the factors that affect the implementation of e-health systems. The use of the NPT [18] can help generate robust explanations of how, why and in what circumstances interventions do or do not work, thus addressing crucial questions relating to, for example, ‘variation in improvement’ [20]. Therefore, we have applied the NPT to the analysis of the implementation of a digital system for the coordination of volunteers in three municipalities to address the research question.

## Methods

### Study design

A case study was designed to follow the implementation of a digital system for collaboration with volunteers and the coordination of activities between healthcare services and volunteer centres. A case study approach is recommended when the purpose is to explore complex interventions in context using various data resources [21]. In addition, in this study, the boundaries between the phenomena of the implementation of the digital system for collaboration and the context were not clear, which also makes case studies an appropriate approach [21]. A single case study was chosen by examining the same issue but in three different units. In this article, the units will be termed as municipalities M1, M2 and M3. Qualitative data collection methods, including interviews and observations, were used to obtain in-depth knowledge about healthcare employees’ experiences with the benefits and barriers in the implementation process. Interviews with healthcare employees and employees from the volunteer centre were conducted in the three municipalities. In addition, three of the authors participated in training sessions and meetings with the employees to follow the implementation process of the system.

### Setting and selection of cases

The current study was related to the Interreg North Sea Region research and innovation project called “*In For Care. Informal care and voluntary assistance: Innovation in service delivery in the North Sea Region*” (Journal-ID: 38–2–12-16). Three municipalities in Southern Norway took part in the project, which involved the implementation of a technology solution aimed at improving the interaction between healthcare and voluntary sectors. Two of the participating municipalities were medium-sized municipalities with 24,000 M1 and 15,000 M2 inhabitants, respectively. The third municipality (M3) was small, with 7000 inhabitants. All municipalities had their own volunteer centres, whose main task was to coordinate volunteer activity. Part of this coordination activity was aimed at and in close cooperation with the municipality’s healthcare and social services.

The present study builds on previous research by the same authors [3], where the need for future digital solutions was mapped. In that research, a codesign workshop gathered healthcare employees, volunteers and relatives to identify the most important features for digital tools. The main objective of the Norwegian partners of the “In For Care” project was to develop a new digital tool. However, the project found that a system offered by a Norwegian vendor met nearly all the identified requirements for volunteer coordination and collaboration. Therefore, this system was acquired and tested in the three municipalities. The system had two modules, one for the volunteer centre and another for the healthcare units the municipality. In the system, there were possibilities for collaboration based on a common activity calendar. The system had a function for employees in the municipality to request voluntary assistance from the volunteer centres. It generated an overview of volunteers and activities. In addition, the system was also designed for volunteers to sign up, sign in, read information about upcoming events and subscribe to tasks.

### Participants

Managers in all three municipalities helped in recruiting by asking employees from the volunteer centre and healthcare services to take part in interviews and observations. All the participants had participated in the implementation and use of the system. The lead author scheduled interviews and observations with these employees. In M1, healthcare professionals were recruited from two nursing homes and two daycentres connected to the nursing homes. In M2, the health care professionals were recruited from home care services and an ambulatory team for mental health and substance abuse treatment. In M3, the only healthcare professional was an employee at the day centre connected to the nursing home. In addition, three of the authors, including the lead author, participated in training sessions and meetings with the employees to follow up on the implementation of the system. See Table 1 for an overview of interviews, observations, training sessions and participants.

**Table 1:** Overview of participants and collected data

Participants / data collection event	Observation of training session with the vendor of the system. All municipalities were present.	Individual interviews before pilot deployment of the system.	Observation of a training session with an external IT expert. All municipalities were present.	Individual interviews and observation of use of the system, after six months of system deployment.	Individual interviews after 12 months of system deployment.
Health care professionals	2 (M1)	1 (M1)	2 (M1)		1 (M1)
		1 (M2)	1 (M2)		1 (M2)
		1 (M3)	1 (M3)		1 (M3)
Municipality managers	1 (M2)	1 (M2)	1 (M2)		
	1 (M3)				
Volunteer centre managers	1 (M2)	1 (M1)	1 (M1)	1 (M1)	1 (M1)
	1 (M3)	1 (M2)	1 (M2)	1 (M2)	1 (M2)
		1 (M3)	1 (M3)		1 (M3)
Volunteer centre employees		1 (M2)	1 (M1)	2 (M2)	
			2 (M2)		
Number of participants	6	8	11	4	6

Number of participants (M1 = municipality 1, M2 = municipality 2, M3 = municipality 3)

### Data collection, interview design and content

**Fig. 1:**
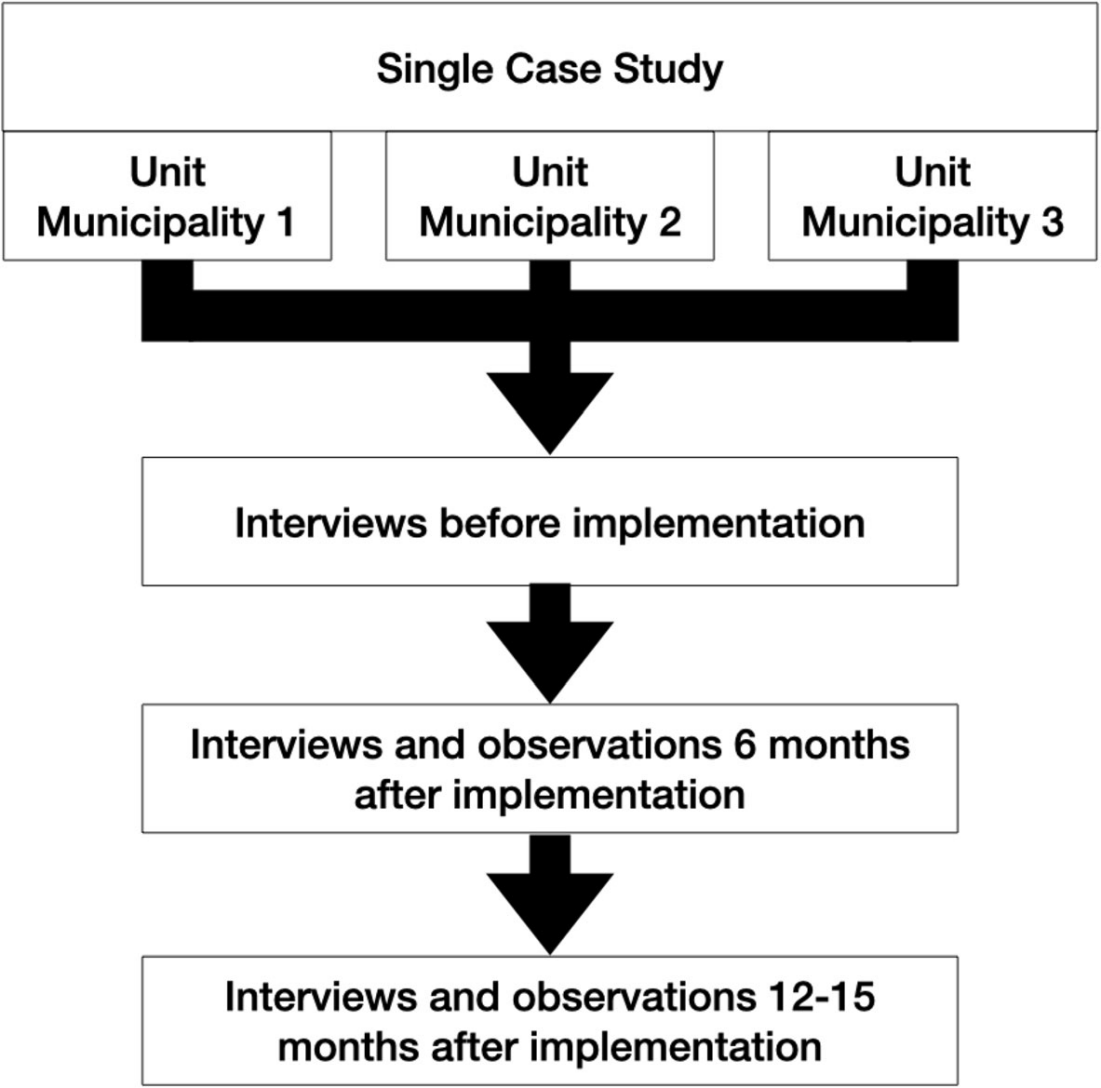
Flow chart

The system was implemented during autumn 2018. Three rounds of interviews were conducted in total (see Fig. 1 for a diagram of the process). The interviews were designed for a longitudinal data collection of the implementation process, knowledge of the user needs, commitment, experiences and improvements (see attached interview guide 1 and interview guide NPT). A first round of interviews with the healthcare employees and employees from volunteer centres was conducted before the deployment of the system (from April to June 2018), except for one after (February 2019) due to challenges with the recruitment of participants. All interviews were conducted by the first author. A semi-structured interview guide was used with questions that addressed how the participants collaborated and coordinated volunteers, what activities they coordinated with volunteers, and follow-up on the practices and expectations of the digital system. A second round of interviews and observations was performed 6 months after the implementation (January 2019). The observations targeted how managers and employees from the volunteer centres used the system. A third and final round of interviews was performed approximately 12 to 15 months after implementation (August to November 2019). The interview guide applied in the last interview round was based on the NPT framework. The guide addressed topics such as participants’ understanding of the system and the reasons for pilot deployment, set up, how they used the system and their first impression of the system when they started using it. In addition, the participants were asked how they collaborated between the municipality and volunteer centre, how the system met their expectations, what critical issues and possible strategies existed and the extent to which they had been supported by the municipality in the deployment process.

The interviews lasted approximately 60 min and were held at the participants’ offices. The interviews were recorded and transcribed verbatim by the first author. In addition, notes were taken during the interviews and observations.

Ethical approval was provided by the Regional Committee for Medical and Health Research Ethics (REK, reference number 2018/1553) and approval from the Norwegian Science Data Services was obtained (reference number 54985/3/HIT).

### Data analysis

All the data were transferred verbatim to NVivo software, version 11 (QSR International Pty Ltd., Melbourne, Australia) for analysis. An inductive thematic analysis was conducted [22] by the main author. The thematic analysis consisted of six phases. The first step was to become familiar with the data (phase 1) before generating initial codes (phase 2), searching for themes (phase 3), reviewing the potential themes (phase 4), renaming and defining the themes (phase 5) and, finally, producing a report (phase 6) [22]. All authors collaborated, discussed and defined themes in phase 5. Further, the data were organised into themes and subthemes based on the categories in the NPT framework. All the data were first analysed separately within the units (municipalities) but also across the different units to better illustrate the case [21].

## Results

Based on the inductive analyses, 11 themes were identified. Further, a deductive analysis according to the four constructs of the NPT was conducted. Two of the themes did not fit the constructs and are therefore grouped under other findings. For an overview of how the analysis was conducted, see Table 2. Given that the interaction of the constructs is dynamic and iterative, a categorisation of the findings aims to provide a systematic overview of the critical issues and strategies for the implementation process. Findings outside the four constructs are also described.

**Table 2:** Overview of the analysis

Participant’s quote	Findings	NPT constructs and other findings
Employee volunteer centre M2: *“The system will give better collaboration between the municipality, volunteer centre and voluntary organisations”.*	• Understanding of needs	Coherence
Employee M1: *“I think the structure is going to be better and it will be easier to keep in touch …*”	• End-user’s expectations	
Observational data showed that the three municipalities had different starting points.	• Collaboration structure	Cognitive participation
Manager from volunteer centre M3: *“You were not free from regular daily tasks to get acquainted with it”.*	• Management’s role	
Employee M3: *“The developer of the system must be responsible for the training”.*	• End-user’s training	Collective action
Employee from volunteer centre M1: *“Several do not have enough computer skills”.*	• User’s experience with the system	
Employee from volunteer centre M2: *“The system helped to improve the structure, and it was easier to contact and coordinate volunteers to assignments”.*	• Realised benefits	Reflexive monitoring
Employee from volunteer centre M2: *“It should have been more intuitive”.*	• System improvement	
Manager from volunteer senctre M1: *“The municipality managers were not clear in organising us [for the implementation], which role we had and which tasks”.*	• Implementation process improvement	
Manager from volunteer centre M2: *“It was time-consuming to set up and make a structure in the system”.*	• Time-consuming process	Other findings
Employee M1: *“It is still important to have a first face-to-face meeting with volunteers to map interests”.*	• Face-to-face communication	

## Coherence

Coherence deals with the employees’ understanding of the aims, objectives and expected benefits of the intervention.

### Understanding of needs

Many of the employees from the municipality and volunteer sector had participated in joint focus group interviews prior to the implementation, where they had discussed the challenges related to cooperation in managing volunteers [3]. In addition, they participated in a workshop where they outlined their needs regarding a digital solution. In the interviews prior to the acquisition of the system, it emerged that the employees believed the system would cover many of the needs that had been discussed in the previous focus groups [3].

The three municipalities decided to buy both modules of the system. The employees from the municipalities thought that the system would contribute towards building a stronger connection with the volunteer centre and a better understanding of how they could use the centre as an asset for their users. They also thought that using the system would save time. One employee said, *“I think it would be a quick way to collaborate, and we need that* [ …] *if someone is busy and you can’t reach them, you have to call later* [ …] *it steals time”.* They expected it would be easier if they could refer patients through a system at the volunteer centre.

### End-user’s expectations

The employees had an understanding that the system could provide better coordination of voluntary services; they thought the system would help them with several tasks that would improve their workday through better systems and structure, a better overview of volunteers and activities, and help in the recruiting and matching processes. The employees provided examples of areas they expected to be improved by the system. They said that some events required registration done via phone and text messages and expected that the new system would improve efficiency by digital means. Furthermore, they employees hoped for a better overview of volunteers and for a better system to follow up with the volunteers.

The employees at the volunteer centres hoped that such a system would help to coordinate the specific requests for volunteer activities. In addition, they thought it would give better availability for the volunteers, a better overview of tasks and provide a way to sign up for events. The employees also expected that the system could support the need for a secure storage of documentation. Further, generate statistics and help with documenting who participated at specific events, which would allow for the monitoring of attendance.

## Cognitive participation

Cognitive participation focuses on the participants’ commitment to drive the implementation forward and the factors that promote and inhibit this commitment.

### Collaboration structure

Despite the employees agreeing that the system would meet many of their needs, the starting point for using the tool was different for the three municipalities. M1 and M3 did not have a structured collaboration between the healthcare services and volunteer centre prior to implementation, while M2 had established a collaborative structure several years ago, where each healthcare unit had its own contact person for the volunteer centre.

### Management’s support

A challenge present in M1 was that the employees did not have a clear understanding of what was expected of them in the implementation process. This was especially true of the employees’ understanding of their role. Because the implementation was part of a project externally funded, much of the responsibility for training the employees was placed on the project, and the managers acted passively. The employees in M1 and M3 experienced that the municipalities’ managers were not present during the implementation process. Therefore, no decisions were made about which healthcare units were going to implement the system and who would be responsible for it. During implementation, the employees from M1 and the volunteer centre had a meeting to determine how they could collaborate, but they did not find a solution. As a result, the system was only implemented at the volunteer centre and not at the municipality healthcare unit. The employees at the volunteer centre used the system for an overview of volunteers and for sending invitations to events. The volunteer centre also unsuccessfully tried to get other volunteer organisations to collaborate with them by using the system. Further, they tried to get volunteers to use the system, but several volunteers were older adults who had little experience with using apps and digital systems.

M2 implemented the system at the volunteer centre and within all their healthcare units, and there the managers were present in the process. Each unit in the municipality healthcare services had an assigned contact person. The employees had a high level of motivation for using the system because they saw the benefits and believed it would meet their needs. All employees involved in the different healthcare units and at the volunteer centre received the necessary time for training. They did not manage to use the system for collaboration with other non-profit organisations (NGOs), but by using the system, they took on the responsibility of coordinating volunteers for a non-profit organisation.

M3 did not fully implement the system during the project period because of a lack of management and role clarification. This may also be related to the municipality being in the process of reorganising and merging with two other municipalities, which introduced uncertainty about which system would be used in the future. Furthermore, several employees were not familiar with digital systems, therefore having little incentive and resources to drive the process forward. A manager from the volunteer centre in M3 said, *“I think we achieved little cooperation. One reason is because there were many meetings about merging with other municipalities. I had limited time to work with it* [the system], *and I am not so familiar with computers”.*

There were some differences between the municipalities in terms of commitment to and follow-up on the implementation. The managers in M1 and M3 were not present during the implementation process, there was no anchoring to municipal strategy, and no extra time was given to become familiar with and start using the system. When the lead author asked whether the municipality had facilitated time for training and using the system, the manager from the nursing home in M1 answered (laughing), *“No, they have not”.* The manager at the day centre in this municipality told us, *“We got to attend to meetings, but you are not redeemed from regular daily tasks to get familiar with it* [the system]*. No, it is something you need to do in your spare time”.*

The manager from the volunteer centre in M1 raised questions about how the managers in the municipality handled the implementation: *“I think the municipality needs to focus on volunteerism; they need to give a position to a volunteer coordinator, not just a task in addition to the other regular tasks* [ …]” *.* Despite little commitment from the managers, the employees helped to spread the word about the system in the municipalities, which triggered the engagement of their colleagues from other departments, believing it could meet their needs.

## Collective action

Collective action is about whether the participants use the system and about the factors promoting or inhibiting its use.

### End-user training

The employees from the three municipalities were offered training both from an IT expert who participated in the “*In For Care”* project and from the vendor of the system. Training with the vendor was performed via videoconference (MS Skype). The employees who participated were satisfied. However, several employees did not have the opportunity to participate and instead got a recording of the video-training session. They reported difficulties with sound and interruptions in the recording. Furthermore, several employees encountered challenges when using the system, but they said that questions were promptly solved by the vendor. It was time-consuming for the employees to prepare to use the system, for training and for collecting consent from volunteers and users that would be registered in the system. Additionally, they found it time-consuming to train other employees and volunteers. They felt that they should have used the system regularly to remember what they had learned, but because they did not have time to do so, they never became completely familiar with it.

### User experience with the system

M2 was the only municipality able to test both modules. The employees at the volunteer centre used the system for an overview of the activities and volunteers and for communication with and coordination for one-to-one tasks. Automatic reminders were sent to the volunteers through the system, which saved time for employees. The employees followed up through phone calls and wrote a log in the system. This was noted as an important feature for managing volunteers. However, the employees at the volunteer centre in M2 met several challenges. Because of their high motivation, they started to register all their volunteers, users, events, tasks and activities in the municipality into the system. Several employees participated in the registration but did not complete it because of interruptions to their work. Afterwards, when the employees sent messages to volunteers with an invitation for events, not everyone received these invitations because their phone number was not registered. This was frustrating for the employees and confusing for the volunteers. The employees were frustrated that they had to spend a significant amount of time finding out which volunteers had incomplete registrations. The employees reflected on this after using the system for a while and felt they had been too enthusiastic and implemented too many tasks and events at once. A manager from the volunteer centre in M2 said: “*We did not have enough time for this, but we had set a whole day in August to work with it. This time we were going to be good and give it a chance; we were going to send out invitations to a kick-off, and we ended up with going home without having completed it”.*

Another challenge was that the system was tailored to support one-to-one activities/tasks, not repetitive tasks, which were the most common. However, the system vendor solved this challenge after requests from this municipality. During the period in which the study was conducted, only a few volunteers in the three municipalities tested the application. Several of the volunteers were old and did not have the computer skills necessary to use a digital system. A manager from the volunteer centre in M1 said: *“There are so many data illiterates among the older adults* [...]*”.* Younger volunteers preferred text messages or e-mail. When the employees in M1 introduced the system to the volunteers, they did not understand why they had to use this system instead of doing their tasks in the traditional way. In M2, the volunteers tested the application but had problems logging in, which created frustration among them and the employees. There were also challenges for those who did not have a smartphone. These volunteers had to make a call to accept a task or invitation to an event that they had already received an invitation for through a text message.

## Reflexive monitoring

The construct of reflexive monitoring is about the participants’ experiences regarding the system’s effectiveness, usefulness and impact on daily practice.

### Realised benefits

The employees who tested the system believed that it contributed to better solutions for collaboration and coordination and improved their working routines. A manager from M2 said: *“At first, I thought it wasn’t good. After a while, I started to like it and I saw the benefits, especially for coordination of volunteers of one-to-one activities”.* The employees in M2 thought it provided a safe environment for documentation storage and reduced the number of paper-based activities. In the long-term, the employees wanted to be paperless. They felt the system was easy for the coordination of volunteers’ activities and events, giving the services a better structure and overview of the volunteers and activities. The employees from the health care units in M2 thought it was easier to communicate and forward messages to the volunteer centre through the system when they were asking for assistance. This created a better information flow than through oral messages, which were easily forgotten. An employee from M2 said: *“I think it* [the system] *was very easy and it felt good to use the computer. The patient said what she/he needed; we added the information details that were important. When I logged out, wow, now I have already been in contact with the volunteer centre, this they will know”.* According to the employees, the system covered several needs, such as the coordination and retention of volunteers, but it did not support recruitment processes. However, the employees highlighted the need for face-to-face contact when recruiting volunteers. Therefore, such meetings before starting as a volunteer helped to map interests and skills and match the volunteers to the right patients. It was also important in retaining volunteers that they be seen and heard and, therefore, this was a priority for employees in M2.

### System improvements

The employees had several suggestions for improving the system. They thought it could be more user friendly and intuitive, with a better connection between the activities, and with an improved logical flow. The manager from the volunteer centre in M1 thought the design was old fashioned; she felt other similar systems had a more appealing design. Currently, different organisations can ask for volunteer assistance through the system and reach out to volunteers to perform specific tasks. However, the employee at the volunteer centre in M2 believed that it should be possible for everyone, including relatives, neighbours and others, to directly ask for voluntary assistance. In addition, they thought a user guide would have been helpful. The employees said that such a digital system might suit larger municipalities better than smaller ones because the larger ones already have a better overview of volunteers. A manager from the volunteer centre M1 said: *“The system is for those people who like to have things structured and organised, those who have many different volunteers”.*

### Implementation process improvements

Employees were asked what kind of long-term expectations they had when using the system. One manager from the volunteer centre in M1 thought there were a few difficulties with the system, but it gave opportunities for developing the services: *“They have planned the system well”.* Furthermore, one employee thought it was important to participate in courses on volunteerism to get a broader understanding of recruitment and retention because this would help them understand the different benefits of using the system.

According to the employees, fully using the system and realising its potential depended on cooperation between organisations, such as volunteer centres, their municipality and volunteer organisations; they felt that this was time-consuming to achieve and that the different units needed to be engaged and committed. Designated contact persons were needed for achieving the potential realisation, such as managers providing guidance and being present. Therefore, according to the employees, politicians and municipality managers should invest their time and effort and be involved in achieving a functional system. They also had to show clarity regarding their expectations of their employees. Furthermore, the employees thought it would have been better to have designated time to set up and use the system. They expressed that different levels of access were necessary for control and to protect privacy.

In M1 and M3, the employees saw benefits, but they were uncertain about what the managers had decided to work further with. In M2, the services had evolved through using the system. However, in the end, they switched to another system from a different vendor because they believed that vendor’s system was a better match with their needs.

## Other findings

### Time-consuming process

Setting up and becoming familiar with the system was time-consuming. It required training for the employees and volunteers who were using the system. The employees also needed a detailed overview of all the volunteers and patients that they were going to register in the system. Further, they needed to obtain a declaration of consent to meet the GDPR data protection requirements. Another important aspect was to maintain confidentiality in the flow of information.

### Face-to-face communication

Despite a system that met several of employees’ needs, face-to-face contact with the volunteers was still important. For instance, a personal first meeting to map interest and match with the right patients was necessary. In addition, having follow-up conversations and showing appreciation for the volunteers motivated them.

## Discussion

The current study has explored the implementation of a digital system for collaboration with and coordination of volunteers in municipal healthcare services in three Norwegian municipalities. The data collected during the implementation process were analysed by using the four main theoretical components/constructs of the NPT. The implementation and intervention of new technologies in healthcare settings are complex [14, 23]; therefore, careful consideration in the planning and execution of the process is needed. Other scholars have studied the implementation of technology in healthcare settings [17, 24–26], and the NPT has been applied for structuring the analysis and results of these studies [13, 14, 23, 27]. Collaboration between public healthcare services using technology has also been previously studied [28–31], showing the importance of achieving understanding, commitment and engagement towards an intervention to succeed [13, 14, 23, 27]. What differentiates our study is that the implementation process involved employees from the public and civil sectors, that is, employees in municipal healthcare units and volunteer centres, providing insights into the implementation of technology that can help different organisations collaborate on the coordination of volunteers. The current research finds that there are several critical issues that need to be considered before and during the implementation, outlining the possible strategies for handling these issues. The current research was longitudinal and followed the process throughout the implementation stages over one-to-one and a half years, looking at the process starting with user needs, ICT system selection, deployment, user training and finally to system refinement. Making sense of the intervention for specific people and how it will affect the tasks and responsibilities for others is a part of the system’s *coherence* [32]. Our findings show positive engagement towards the system in all three municipalities. The prior participation in codesign workshops in two of the municipalities for mapping needs provided a common understanding that a new digital solution for collaboration was needed. Hence, the employees showed enthusiasm for implementing and testing the system. User involvement and design in accordance with the user’s specific needs were crucial in the implementation process, which is in line with previous studies [17], showing that user involvement can promote coherence among participants and increase contributions towards a common understanding of the purpose of the intervention [23, 27].

*Cognitive participation* or engagement and commitment were slightly different across the three municipalities*.* The employees in M1 and M3 expected that their managers would have provided guidance and assigned responsibility for the implementation process, but their managers were absent. They did not receive any information about their roles; therefore, they were hesitant during the implementation process. This fact created a high level of uncertainty regarding responsibility, which in turn influenced the commitment negatively during the preparation phase.

In M2, a structure for collaboration was established prior to the intervention, making the implementation process more agile. The structure existence confirms a prioritisation shared among leadership, one that facilitates the coordination of volunteers from the top-down. This seems to have contributed to the commitment of the employees.

Several meetings were held in M1 and M3 to build a structure for collaboration, but these meetings never reached an agreement. Therefore, none of the employees took the responsibility to move forward with the implementation process. This finding is in line with prior studies showing that there are often key people and champions who have the capability to promote the utilisation of new digital systems in healthcare services, as opposed to reticent colleagues who will not move the project forward [19, 32]. The key people/champions can involve others and generate commitment, whereas colleagues with negative attitudes can jeopardise other employees’ commitment needed to make a system work and, thus, impede the implementation [19]. In M1 and M3, the employees complained about the absence of leadership, lack of expectations, role clarifications and training time, and we believe this hampered commitment.

Policy making can be done as a top-down or bottom-up approach [33], where the top-down approach takes policies that are designed/defined in government spaces as a starting point. Furthermore, the top-down approach emphasises the contextual integration of a system and the extent of management and resourcing [19]. The findings from our study show that leadership was crucial for the process: the employees needed clarification of their roles and expectations, and this influenced their commitment. Another aspect can be the organisation’s maturity [34, 35] to implement such a digital system that requires collaboration. The lack of structure for this in M1 and M2 affected the process, in addition to the lack of management in these two municipalities.

*Collective action* is the actual process of implementation [32]. Because of the lack of management and/or key people to drive the intervention forward, the system was not implemented in M3 and was only enacted at the volunteer centre in M1. M2 implemented the system both at the volunteer centre and in each municipality healthcare unit. The findings of the present study show that the preparation was time-consuming and that the employees depended on receiving enough time to train, get acquainted with, set up and use the system. The employees expected their managers to give them directions, and this did not happen in M1 and M3; hence, the collective action was hindered. The implementation of new technology includes the dissemination of information in the preparation phase [19]. However, the implementation process in M2 was not completely successful either. In the first phase of the process, the employees had high levels of motivation. They ended up registering too much information in a short amount of time but became confused because the registration was incomplete. This led to frustration among the employees and a general perception that the system was not working properly. This can be related to relational integration, or the knowledge work, that the participants create to achieve accountability and maintain confidence in a set of practices, for example, access to relevant information [32].

Computer skills were found to be an important aspect in our study, both for employees and the volunteers using the system. Several informants reported a low data literacy level and hesitated to use the system. Low computer literacy has an impact on skill set workability [32, 36], which will constrain the process regarding people’s roles and responsibilities [32]. Some employees do not see working with computers as their core task and are hesitant to use new systems [37]. Other studies [38] have also found that most volunteers are older adults and have a wide variation regarding their technology skills. There was then a need for time and effort to train the volunteers who showed resistance in using the technology, which is in line with what was reported in [9]. The current study found several factors related to *reflexive monitoring* after using the system. Appraisal of the intervention is both individual and collective, and it can be evaluated informally and formally [32, 39] in relation to critical issues and possible strategies. Basically, all the employees saw the benefits of the system, and all three municipalities bought the two modules. However, only employees in M2 tested the municipality module, and the employees from the healthcare units found it easy to send questions about patients who needed volunteer assistance. Using the system saved time for municipality employees, who had a busy working schedule.

The quality of the system itself also affected the implementation process, and there are findings from previous research on a similar type of system. What a digital solution was tested for the same purpose, a success factor with this application was that the volunteer pool expanded with a diversity of volunteers, for example, age, gender, specific skills and education [9]. This influenced the coordination practices and created more rigorous recruitment. The system in our study was not used for recruitment, perhaps because of the short timeline of the current study. However, we found that the new system resulted in a decreased administrative burden for acquiring an overview and generating statistics [9]. Another finding related to one of the downsides, where the system did not ensure the accountability and quality of the volunteers [9]. Face-to-face contact was still important [3]. In our study, we applied the NPT to understand and analyse our findings. The benefits of using a theory to analyse the findings is a generalisable framework that can be applied across different settings and individuals, helping understand the barriers to implementation [14]. Using the NPT has helped to explain the intervention work by looking at the implementation process at an early point, but also when it was embedded into practice. The four constructs of the NPT are not linear but share dynamic relationships internally with each other and with the wider context of the intervention, such as the organisational context, structures, social norms, group processes and conventions; furthermore, the constructs overlap each other [14, 40–42]. Analysing and connecting our data to the right construct was challenging because of the nonlinear process and the overlap. Other researchers have had the same experiences [43, 44]. However, analysing the data inductively and looking for themes first gave us in-depth comprehension of the data. It is complex to analyse the data using a deductive approach to directly connect it to the four constructs; this problem is less evident when using an inductive approach [41].

### Strengths and limitations

The municipalities were all part of the “*In For Care”* project and were testing the system as part of the project. This may have required resources and knowledge that might not have been foreseen or initially included in the municipalities’ strategies. The credibility of this scientific qualitative research may be assessed based on its validity, reliability and transferability. The three concepts are underpinned by critical reflection [45]. Regarding its validity, the study responded to what it addresses [46], that the research has sought to study a digitalisation of municipal healthcare collaboration with volunteers through the follow-up of the implementation process with a new municipal service. The most natural way was to question the people involved working in the same municipalities. The informants were very helpful, and the close relationship built with them throughout the whole process helped to sincerely address the questions asked in the interviews. The reliability of the study was supported by presenting quotes from the interviews (see Table 2), highlighting informants’ own voices. The transferability of a study depends on whether the findings can be applicable in different contexts.

A possible limitation is that interviews and the initial coding were conducted by one author only. However, all authors took part in the analysis, and in quality assurance of the data collection and the coding. The research has provided valuable knowledge not only on the findings, but also on how to follow-up an implementation process in municipal settings from the beginning until the end, reporting all the steps of the process. These facts can not only help to improve practices in municipal settings in other regions and countries, but also assist implementation in other types of organisations.

### Recommendations

#### Implications for practice

A system for collaboration with and coordination of volunteers can provide several benefits for a better overview, easier communication, and improved information exchange. However, our study has shown that several aspects must be present during the implementation process. Management’s presence is crucial in the implementation of a digital system for the collaboration with and coordination of volunteers, especially when different organisations and municipality units are collaborating on the assignment. Further, management should play a proactive role and help employees obtain an understanding of how the intervention should be integrated or change contemporary practice. Management needs to engage and lead the employees regarding preparation, expectations, roles and responsibility for motivation throughout the process. A structure for collaboration between the municipality units and volunteer centres would also be beneficial. In addition, time for training, setting up and incorporating the system into daily practice is needed. In this process, it is important to consider the diversity of employees’ computer literacy skills and assign additional time to train and build up the necessary motivation and confidence among the employees for the implementation process. For the same purpose, it is important to start with an appropriate level of ambition, meaningfully selecting which part of a digital system to start, set up, use and with which to become familiar. Also, it is important to consider which volunteers can be trained in the system for successful integration.

#### Implications for research

Further research is needed regarding the process of implementing a digital system for collaboration and coordination because there is little research in this area. Qualitative research could shed light on the critical issues and potential strategies in the implementation process. In addition, there is a need for research on the effects of using the system on the experiences regarding the recruitment, collaboration with, coordination of, training and retention of volunteers. We also recommend quantitative research on these issues, which can provide statistics on the recruitment, satisfaction and retention of volunteers.

## Conclusion

The use of the NPT and the four constructs in the current study gave direction and guidelines for the authors to organise the data, helping us understand the implementation process for the collaboration with and coordination of volunteers. Several aspects related to preparing for the implementation process of the system are key issues for consideration. These are related to municipality’s strategy, leadership directions, the employees’ engagement and commitment, training and additional time for set up and use of the system are contributing factors for the implementation. The present study has provided results on how a system can contribute to improving the structural collaboration between organisations in the coordination of volunteers who need to work on different tasks, hence obtaining a better overview, systematisation and secure storage of documentation. However, face-to-face contact is still important for the coordination of volunteers, matching them with appropriate tasks and thereby retaining them.

## Supplementary Information

**Additional file 1.** Interview guide (round 1 and 2).

**Additional file 2.** Interview guide NPT (round 3).

## Data Availability

The datasets generated and analysed during the current study are not publicly available due to anonymity purposes and to safeguard the participants, confidentiality, but the transcriptions are available from the corresponding author on reasonable request.

## Abbreviations

NPT: Normalisation process theory
NGO: Non-profit organisation
GDPR: General data protection regulation
