# Supplementary material for: Digitalisation of municipal healthcare collaboration with volunteers: a case study applying normalization process theory
Source: BMC Health Serv Res. 2021 May 1;21:410. doi: 10.1186/s12913-021-06429-w (PMC8088692; doi:10.1186/s12913-021-06429-w)
Supplement: Supplementary file 1 — Additional file 1. Interview guide (round 1 and 2). [file 12913_2021_6429_MOESM1_ESM.docx]

# **Interviewguide (round 1)**

Can you describe a typical working week

What tasks do you carry out when working with volunteers?

How do you coordinate the volunteers? (use of phone, SMS, email)

- Can you describe the amount of time you spend in the coordination of volunteers?
- What is most time consuming task/aspect?

Which activities do you coordinate?

How do you carry out the first conversation and the follow-up conversation with volunteers?

What kind of information do you gather and where/how do you store it?

Can you show the system (excel, word) where you store the information related to volunteers?

What are your expectations towards a system for coordination and collaboration with volunteers?

- How do you think it can improve your working day?
