# Supplementary material for: Digitalisation of municipal healthcare collaboration with volunteers: a case study applying normalization process theory
Source: BMC Health Serv Res. 2021 May 1;21:410. doi: 10.1186/s12913-021-06429-w (PMC8088692; doi:10.1186/s12913-021-06429-w)
Supplement: Supplementary file 2 — Additional file 2. Interview guide NPT (round 3). [file 12913_2021_6429_MOESM2_ESM.docx]

# **Interviewguide NPT (round 2 and 3)**

**Coherence**: participant understanding of why the digital system Frida has been implemented

Why this system?

Do you understand the purpose of using the system?

**Cognitive participation:** staff engagement and commitment to Frida

Can you explain how do you use the system? Is it possible to use the system for additional/other tasks? Would you like to use the system in a wider context? (Which activities do you coordinate in the system?)

What was your first thought when using the system? What were the thoughts inside the municipal department?

**Collective action:** Frida usage by participants

How did you set up the system? How does the system support the coordination of volunteers?

- Which participants are involved from the municipality and the volunteer centre?
- How do you collaborate? (structure)
- Did you need to do any changes in the current structure? How is this different from before?

How does the system affect your way of working?

Have there been any challenges in using the system?

- How can these possibly be improved?

How is the system being followed up by other employees in the municipality/volunteer centre?

- Are you dependent on others’ use to benefit from the system?
- Has the collaboration been changed? Example, the use of activity calendar and events

How is the documentation storage, is it different?

- Do you still use paper-based contracts to get overview of the volunteers?
- How does the system support requirements for handling personal data?

**Reflexive monitoring:** staff appraisal of Frida

How do you experience that the municipality management facilitates usage of the system?

- Support in the implementation process
- Time and resources for training

How does the system meet your/municipality/volunteer centre expectations, what are the benefits and the improving factors?

- In which way and what areas has the system supported your working day?
- Does it fit the priorities?
- Does it cover the municipality/volunteer centre needs?

Can you say anything about what kind of benefits there are using the system?

- Efficiently
- Collaboration with other organizations
- Collaboration with volunteers
